# Maternal Environment Influences Cocaine Intake in Adulthood in a Genotype-Dependent Manner

**DOI:** 10.1371/journal.pone.0002245

**Published:** 2008-05-21

**Authors:** Rixt van der Veen, Muriel Koehl, D. Nora Abrous, E. Ronald de Kloet, Pier-Vincenzo Piazza, Véronique Deroche-Gamonet

**Affiliations:** 1 U862, Institut National de la Santé Et de la Recherche Médicale (INSERM), Bordeaux, France; 2 Division of Medical Pharmacology, Leiden, Amsterdam Center of Drug Research, Leiden University Medical Center (LACDR/LUMC), Leiden, The Netherlands; Vrije Universiteit Amsterdam, Netherlands

## Abstract

**Background:**

Accumulating epidemiological evidence points to the role of genetic background as a modulator of the capacity of adverse early experiences to give rise to mental illness. However, direct evidence of such gene-environment interaction in the context of substance abuse is scarce. In the present study we investigated whether the impact of early life experiences on cocaine intake in adulthood depends on genetic background. In addition, we studied other behavioral dimensions associated with drug abuse, i.e. anxiety- and depression-related behaviors.

**Methodology/Principal Findings:**

For this purpose, we manipulated the maternal environment of two inbred mouse strains, the C57BL/6J and DBA/2J by fostering them with non-related mothers, i.e. the C3H/HeN and AKR strains. These mother strains show respectively high and low pup-oriented behavior. As adults, C57BL/6J and DBA/2J were tested either for cocaine intravenous self-administration or in the elevated plus-maze and forced swim test (FST). We found that the impact of maternal environment on cocaine use and a depression-related behavior depends upon genotype, as cocaine self-administration and behavior in the FST were influenced by maternal environment in DBA/2J, but not in C57BL/6J mice. Anxiety was not influenced by maternal environment in either strain.

**Conclusions/Significance:**

Our experimental approach could contribute to the identification of the psychobiological factors determining the susceptibility or the resilience of certain individuals to develop psychopathologies.

## Introduction

Clinical and epidemiological studies point to an important role of adverse early experiences in the vulnerability to a variety of psychiatric disorders in adulthood. Family environment in particular would affect the long-term neurobiological and psychosocial development of the offspring and modulate the vulnerability to mood-, anxiety-, psychosis- or drug use-related disorders [1]–[6]. But, although their influence may be strong and pervasive, early experiences rarely determine the nature and outcome of the psychopathology [7], [8]. Indeed, large individual differences exist in susceptibility to the impact of early life events on health and behavior.

Numerous twin and adoption studies have demonstrated a gene-environment interaction in the development of psychiatric disorders [9]–[12]. Moreover, accumulating epidemiological evidence suggest that genetic background can modulate the capacity of an environmental risk factor to give rise to mental illness [13], [14]. In particular, functional polymorphisms were found to modulate the influence of adverse early experiences on antisocial disorders [15], [16], schizophrenia [17] and depression [18]. In contrast, direct evidence of gene-early environment interactions in substance abuse disorders is more scarce and mostly concerns alcohol abuse [19], [20].

The accessibility to (illegal) drugs, drug effects and the great individual variability in intake makes epidemiological studies on substance abuse particularly difficult. As an alternative to human studies, interesting animal models exist for many aspects of substance use and abuse. In particular intravenous self-administration (SA) is considered as one of the best animal models of human psychopathology that has high predictive validity for detecting compounds with an abuse potential in humans [21]. Rodent studies using this model suggest that also in the etiology of cocaine abuse, gene-environment interactions could play a role. That is, different adult environmental experiences were shown to influence cocaine SA in a gene-dependent manner [22], [23]. However, it remains to be determined whether long-lasting changes induced by early life experiences can also influence cocaine SA in a genotype-dependent manner.

In the present study we investigated whether the impact of early life environment on cocaine SA in adulthood depends on the genetic background. For this purpose, we manipulated the maternal environment of two inbred mouse strains, the widely used C57BL/6J (C57) and DBA/2J (DBA) by fostering them with non-related mothers. A critical component of the maternal environment is constituted by the behavior of the mother. We therefore chose two strains showing respectively high and low pup-oriented behavior, i.e. the C3H/HeN (C3H) and AKR strains [24]. For reference, we also included mice of both strains raised by their biological mother. In adulthood, mice were tested on intravenous cocaine SA. In parallel with these SA experiments, in a separate set of animals, we investigated whether genotype influenced the impact of maternal environment on anxiety- (elevated plus-maze) and depression- (forced swim test) related behaviors; two dimensions potentially associated with the vulnerability to cocaine abuse [25], [26].

We show that the impact of early life experiences on cocaine use in adulthood is dependent on the genotype, as we found the DBA strain sensitive and the C57 strain resistant to the influence of maternal environment on cocaine intake in adulthood. Additional behavioral characterization revealed that the alterations in cocaine-taking behavior observed in the DBA strain were accompanied with alterations in depression-related behavior while anxiety was not influenced. These results demonstrate a strong gene-environment interaction during the early life period that affects psychopathology-related behaviors in adulthood.

## Materials and Methods

### Subjects

All mice used in the experiments were bred in our animal facilities. Three of the original strains (C57BL/6JOlaHsd, AKR/OlaHsd, C3H/HeNHsd) were obtained from Harlan (Gannat, France), the DBA2JIco mice were obtained from Charles River Laboratories (Arbresle, France). Animals were kept in our temperature (23°C) and humidity (60%) controlled animal facilities. Food and water were available *ad libitum*. All experiments were conducted in accordance with the European Communities Council Directive of 24 November 1986 (86/609/EEC).

### Breeding and cross-fostering

A 14h light/10h dark cycle was installed (lights on from 8h to 22h) as is common in reproductive facilities. In an individual mouse cage (29×11×13 cm), each female was paired with a male of her own strain that was removed from the cage when pregnancy was ascertained by visual observation and body weight recording. Cross-fostering was conducted between 4–7 hours after both biological and adoptive dams had given birth. The procedure consisted of removing the biological mother, weighing and sexing all the pups, culling the litter to 5–8 pups, placing the litter in an individual mouse cage containing bedding of the adoptive mother, and finally placing the adoptive mother in the cage. This cage contained a transparent Plexiglas separation with a small hole creating a nest compartment that occupied one-third of the cage. Culled litters contained a balanced amount of males and females. The whole procedure lasted on average 2 min, and never took more than 4 min. Biological litters were treated as cross-fostered litters, but were returned to their original mother.

### Maternal behavior

Cages were placed in sound safe video-equipped chambers equipped with infra-red cameras to record maternal behavior (Imetronic, Pessac, France). Continuous recordings of maternal behavior were made over 23h periods on postnatal days (PD) 2, 4, 6 and 9. Recordings started at 8h. Only dams with litters between 10h and 24h of age at the start of recording on PD2 were included in behavioral scoring (AKR mothers: *n* = 9 dams for each pup strain, C3H mothers: *n* = 7 dams for each pup strain, bio mothers: *n* = 15 for C57 dams and *n* = 8 for DBA dams). Behavior was scored off line by an experimenter unaware of the experimental groups using The Observer® (Noldus Information Technology, Wageningen, The Netherlands). Six behaviors were scored. *Pup licking:* Licking any part of the pups' body, including anogenital parts. *Nursing posture:* Immobile arched-back posture over the pups, with the abdomen actively elevated from the floor and pups attached to the nipples. *Nest reorganizing:* Nest disturbance and pup scattering by a push forward movement of the mothers' head in the sawdust. *Self grooming on the nest:* Self grooming while being in contact with the pups. *‘Passive’ nest presence:* Being in contact with the pups without showing any of the above mentioned behaviors. *Out of nest:* Not being in physical contact with the pups. An observation was made every 2.5 min. Detailed analysis of maternal behavior is described in an earlier study [24].

### Drugs

Cocaine (Coopération Pharmaceutique Française, Bordeaux, France) was dissolved in 0.9% NaCl.

### Catheters

Catheters were made from 6 cm soft silastic tubing (i.d. = 0.30 mm, o.d. = 0.64 mm; Dow Corning Corp., Midland, Michigan, USA), fitted to a 22G steel cannula (Plastics One Inc., Roanoke, VA, USA) bent at a right angle. The cannula was anchored with dental cement onto the plastic part of a 26G needle and provided with a small piece of nylon mesh for support.

### Surgery

Surgery was performed under ketamine (80 mg/kg, Imalgène®)/xylazine (16 mg/kg, Rompun®) anesthesia as previously described [23], [27]. During surgery, mice were placed on a heat-pad (30°C). The catheter was inserted in the right jugular vein, until the tip reached the level of the right atrium, it was attached to the vein around a little dab of silicone on the tubing. The distal end was passed subcutaneously over the shoulder to be attached in the mid-scapular region. After surgery mice were placed in a heat-box (27°C) until they woke up from anesthesia. To prevent infection animals received daily ip injections of a 0.05 ml gentamicine solution (11.4 mg/ml, Gentalline®) during four days. Throughout the experiment, catheters were flushed daily with a saline solution containing heparin (30 IU/ml).

### Intravenous self-administration apparatus

The intravenous SA setup (Imetronic, Pessac, France) consisted of 16 chambers made of Plexiglas and metal. Each chamber (18×11×15 cm) was located within a larger exterior opaque box equipped with exhaust fans that assured air renewal and masked background noise. In the SA chamber, the intravenous catheter of the animal was connected to a pump-driven syringe (infusion speed: 20 µl/sec). Two holes (Ø 8.5 mm) were located at opposite sides of the chamber at 2 cm from the grid floor. A white cue light (Ø 2 mm) was located 3 cm above each hole. Experimental contingencies were controlled and data collected with a computerized system (Imetronic, Pessac, France).

### Elevated plus maze (EPM)

The apparatus was made of Plexiglas and consisted of a plus-shaped platform elevated 116 cm above the floor. Two of the opposing arms (30×8 cm) were enclosed by 17 cm high transparent walls (closed arms) whereas the other two arms had no walls (open arms). The floor of the maze was covered with black Plexiglas. At the start of the test, the animal was placed at the crossing of the four arms, facing an open arm. The animal could move freely over the maze for 5 min. A camera connected to a computerized tracking system (©VideoTrack, Viewpoint) allowed to measure entries into open and closed arms and time spent in each compartment.

### Forced swim test (FST)

The FST was performed according to the method of Porsolt [28]. Mice were placed individually in a glass cylinder (height 25 cm; Ø 18 cm) filled with 22 °C water to a depth of 15 cm. Behavior was recorded for 6 min with a camera placed to give a side view of the cylinder. The duration of total immobility was scored off-line by an experimenter unaware of the experimental groups. A mouse was judged to be immobile when it remained floating, in an upright position, making only small movements to keep its head above the water.

### Procedures

**Figure 1:**
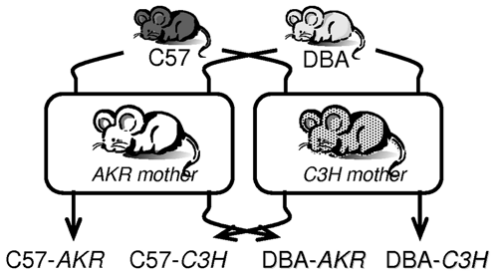
Experimental set-up. Manipulation of the early environment by fostering pups of the C57 and DBA strain with mothers of the AKR or C3H strain.

Pups were weaned and weighed at 21 days of age and placed together with same sex group mates, 4–6 animals per cage (37×21×14 cm). At 10 weeks of age the animals were individually housed under a standard 12h light/dark cycle (lights on from 8h to 20h). Six experimental groups were available for measuring adult behavior (figure 1): C57 and DBA mice raised by mothers of the AKR strain (C57-*AKR* and DBA-*AKR*), C57 and DBA mice raised by mothers of the C3H strain (C57-*C3H* and DBA-*C3H*), and C57 and DBA raised by their biological mothers (C57-*bio* and DBA-*bio*). Since animals from these last two groups have an isogenic mother and are not fostered, they can not be considered as fully valid control groups and thus cannot be included in the analysis of the impact of maternal environment. They can, however, be used as reference groups providing indications of the change that might occur in behavior from mice of each strain raised by one or the other foster mother. As such, they will appear in the background of the graphs. At three months of age, animals from the six experimental groups were attributed to an experiment (1 or 2) and behavioral testing was performed between 3 and 5 months of age.

#### Experiment 1: Cocaine self-administration

**Figure 2:**
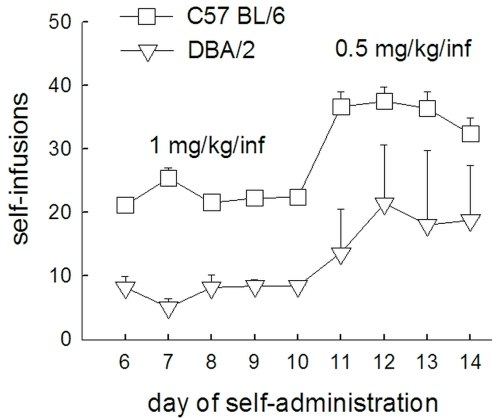
Cocaine self-administration in C57 and DBA mice in a study where both strains acquired at 1 mg/kg/infusion. The graph shows the number of self-infusion on the last 5 days of acquisition (day 6–10) and the transition to a 0.5 mg/kg/infusion dose (day 11–14). At the 0.5 mg/kg dose, DBA mice show a number of self-infusions that is comparable to that of the C57 mice at the 1 mg/kg/infusion dose. Symbols represent mean±SEM. C57 (squares) *n* = 8, DBA (triangles) *n* = 5.

For a first set of animals (n = 12 per experimental group), the light/dark cycle was inverted (lights off from 8h to 20h) and mice were given a three weeks acclimatization period. Then catheterization took place followed by 4–6 days of recovery before the start of cocaine SA. Sixteen SA cages were available, which resulted in three SA sessions per day, the first session starting 1.5 hours after lights off. Animals from the different experimental groups were randomly attributed to a session, each individual animal being tested at the same time each day. When the animal introduced its nose into one of the holes (the active device), this turned on the cue light and subsequently switched on the infusion pump (1 sec cue light alone followed by 1 sec cue light plus infusion). Nose-pokes in the other hole (the inactive device) had no scheduled consequences. A fixed ratio 1 (FR1) schedule was applied throughout the experiment, meaning that one nose-poke was necessary to obtain an infusion of cocaine. Each infusion was followed by a 9 sec time-out period during which cocaine was not available. Mice were allowed to acquire cocaine SA during daily 90 min sessions. In an earlier study [23] we identified a difference in dose sensitivity for cocaine between the C57 and DBA strains; the latter strain having a dose-response curve shifted to the left. In order to obtain a comparable number of infusions at acquisition, C57 mice acquired at a dose of 1mg/kg/infusion and DBA mice at a dose of 0.5 mg/kg/infusion (see figure 2 for data supporting this choice). Criteria for acquisition of cocaine SA were defined by a stable number of self-infusions over at least three consecutive sessions (±20%) and at least 75% responding for the active hole. After 10 days of SA all animals reached these criteria. Following acquisition, a dose-response study was performed. Cocaine dose was gradually diminished over sessions (0.5, 0.25, 0.125, 0.0625 mg/kg/infusion for C57 and 0.25, 0.125, 0.0625, 0.0313 mg/kg/infusion for DBA), maintaining each dose until the animal reached at least two days of stable intake (±20 %). Considering the results, each strain was tested for an extra seventh dose (2 mg/kg/infusion for C57 and 0.0156 mg/kg/infusion for DBA) in order to obtain a bell-shaped dose-response curve for both strains. Finally, both strains were tested for saline during 5 days. Because of blocked or leaking catheters, some animals were taken out of the experiment. Only animals that completed SA were included in the analyses (C57-*AKR n* = 5, C57-*C3H n* = 8, C57-*bio n* = 8, DBA-*AKR n* = 8, DBA-*C3H n* = 11, DBA-*bio n* = 10).

#### Experiment 2: Elevated plus maze and forced swim test

A second set of animals (C57-*AKR n* = 12, C57-*C3H n* = 14, C57-*bio n* = 14, DBA-*AKR n* = 8, DBA-*C3H n* = 10, DBA-*bio n* = 11), bred together with the animals used in experiment 1, was tested on the EPM and the FST successively. Both tests were performed during the light phase (starting 1.5 hours after lights on) under dim light conditions (90 lux). Four weeks separated the two tests.

### Data analysis

Analyses were performed using Statistica 6.0© (StatSoft Inc). *Maternal behavior:* Maternal behavior differences between AKR and C3H mothers were globally analyzed with bifactorial ANOVAs using pup strain and mother strain as independent factors. To situate the behavior of these foster mothers relative to that of biological mothers, within each pup strain, one-way ANOVAs were performed with mother strain (C3H, AKR and bio mother) as independent factor. *Bodyweight:* Bodyweight differences at birth of pups attributed to the different experimental groups were analyzed within each strain using one-way ANOVAs. The influence of mother strain on bodyweight at weaning and adulthood was analyzed with a repeated measures ANOVA using mother strain and pup strain as independent variables and age as dependent variable. To situate the reference group (*-bio*) within each pup strain, repeated measures ANOVAs were performed. *Self-administration*: To verify recognition of the active hole in the acquisition phase of SA, and recognition of the different drug doses in the dose-response test, a repeated measures ANOVA was performed within each experimental group, using either hole and day (acquisition) or dose (dose-response) as dependent variables. Within each pup strain, group differences in responding at the acquisition endpoint (mean of the last three acquisition days) were analyzed with unpaired two-tailed t-tests. The reference groups (*-bio*) were situated using one-way ANOVAs. Group differences in responding during the 10-day acquisition period and during the dose-response test were analyzed with repeated measures ANOVAs, where mother strain served as an independent factor and day or dose as dependent variable. In the dose-response test, we considered the mean of the last two days (stable intake) for each dose. Within each pup strain, the reference group (*-bio*) was situated using repeated measures ANOVAs, where mother strain served as an independent factor and day or dose as dependent variables. *Elevated plus maze and forced swim test*: Mother strain effect on behavior in the EPM and FST was globally analyzed with bifactorial ANOVAs using mother strain and pup strain as independent variables. Within each pup strain, the reference group (-*bio*) was situated using a one-way ANOVA. A significance level of p<0.05 was used for all statistical analyses. Whenever appropriate, a Newman-Keuls post-hoc test was performed.

## Results

### Maternal behavior of foster mothers (figure 3)

Maternal behavior differences between AKR and C3H mothers were globally analyzed including both pup strains. Then, for each pup strain, the behavior of the adoptive mothers was compared to that of biological mothers.

**Figure 3:**
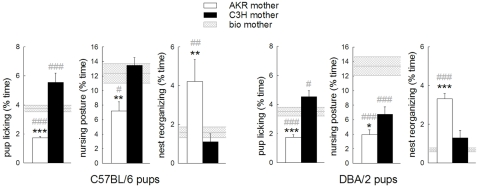
Maternal behavior displayed by AKR and C3H mothers toward C57 and DBA pups. Graphs show the percentage of time spent in pup licking, nursing posture and nest reorganizing activity with C57 pups (left panels) and DBA pups (right panels) averaged over 4 postnatal days (PD 2, 4, 6 and 9). Behavior of the reference mothers (bio mothers) is shown as horizontal hatched bands. Bars represent mean±SEM. AKR mothers (white bars) *n* = 9 dams for each pup strain. C3H mothers (black bars) *n* = 7 dams for each pup strain. Bio mothers (hatched bands) *n* = 15 for C57 dams and *n* = 8 for DBA dams. * p<0.05, ** p<0.01 and *** p<0.001 compared to C3H mothers. # p<0.05, ## p<0.01 and ### p<0.001 compared to bio mothers.

Maternal behavior of AKR and C3H dams was shown to be very different, irrespective of the pup strain raised (see figure 3 for pup-oriented behaviors). C3H dams showed more pup licking (mother strain effect F(1,28) = 97.7 p<0.001, mother strain*pup strain F(1,28) = 2.07 p = ns), more nursing posture (mother strain effect F(1,28) = 21.4 p<0.001, mother strain*pup strain F(1,28) = 3.14 p = ns) and less nest reorganizing (mother strain effect F(1,28) = 14.7 p<0.001, mother strain*pup strain F(1,28) = 0.69 p = ns) compared to AKR dams. Although both foster mothers spend an equal amount of time in contact with the pups, AKR mothers spent more time in self-grooming on the nest and ‘passive’ nest presence, two behaviors that are less directly pup-oriented (graphs not shown). Both mother strains showed less nursing with DBA pups than with C57 pups (pup strain effect F(1,28) = 26.0 p<0.001, mother strain*pup strain F(1,28) = 3.14 p = ns).

The behavior of biological C57 mothers (hatched bands in left panels of figure 3) was situated in-between the foster mothers for pup licking (mother strain effect F(2,28) = 31.6 p<0.001 with bio>AKR, bio<C3H and AKR<C3H, all p<0.001). Considering nursing posture and nest reorganizing, behavior of biological C57 mothers was comparable to that of C3H foster mothers (*Nursing posture:* mother strain effect F(2,28) = 5.7 p<0.01 with bio>AKR p<0.05, bio≈C3H and AKR<C3H p<0.01, *Nest reorganizing*: mother strain effect F(2,28) = 6.87 p<0.01 with bio<AKR p<0.01, bio≈C3H and AKR>C3H p<0.01).

The behavior of biological DBA mothers (hatched bands in right panels of figure 3) was equally situated in-between the foster mothers for pup licking (mother strain effect F (2,21) = 25.6 p<0.001 with bio>AKR p<0.001, bio <C3H p<0.05 and AKR<C3H p<0.001). Considering nursing posture, biological DBA mothers showed higher levels compared to both foster mothers (mother strain effect F(2,21) = 27.9 p<0.001 with bio>AKR p<0.001, bio>C3H p<0.001 and AKR<C3H P<0.05). The nest reorganizing behavior of biological DBA mothers was comparable to that of C3H foster mothers (mother strain effect F (2,21) = 32.7 p<0.001 with bio<AKR p<0.001, bio≈C3H and AKR>C3H p<0.001).

### Bodyweight of offspring (table 1)

Bodyweight at birth did not differ between pups attributed to the two different maternal environments or pups that stayed with their biological mother (mother effect C57 F(2,73) = 1.85 and DBA F(2,62) = 0.71, both p = ns).

**Table 1:** The impact of maternal environment on bodyweight.

Pup strain	Maternal environment	n	BW (g) at birth	BW (g) at weaning	BW (g) at 16 weeks
C57	AKR mother	24	1.39±0.02	7.54±0.23	26.92±0.27
	C3H mother	26	1.35±0.02	10.31±0.25	29.46±0.36
	C57 bio mother	26	1.34±0.02	9.08±0.20	29.65±0.27
DBA	AKR mother	20	1.31±0.03	7.45±0.21	26.30±0.29
	C3H mother	22	1.28±0.03	8.64±0.27	27.18±0.31
	DBA bio mother	23	1.32±0.03	7.30±0.23	26.04±0.35

The table shows bodyweight (BW) data at birth, weaning and 16 weeks of age of male C57 and DBA offspring raised by an AKR, a C3H or their biological mother. Values represent mean±SEM.

When comparing the influence of being raised by an AKR or a C3H mother on bodyweight, it appeared that pup strains were differentially affected (mother strain*pup strain F(1,88) = 10.46 p<0.01). This effect was comparable at weaning and adulthood (age*mother strain*pup strain F(1,88) = 0.02 p = ns). Although it was found that being raised by an AKR mother gives a lower bodyweight compared to being raised by a C3H mother for both C57 (p<0.001) and DBA (p<0.01), the influence of mother strain on bodyweight was more important in C57 mice.

The bodyweight of C57 mice raised by their biological mother, was differentially situated at weaning and in adulthood (age*mother strain F(2,73) = 11.35 p<0.001). At weaning C57-*bio* were situated in-between C57-*AKR* and C57-*C3H* (C57-*bio*>C57-*AKR* p<0.01 and bio<C57-*C3H* p<0.05). In adulthood, C57-*bio* mice continued to be heavier than C57-*AKR* mice (p<0.001), but now showed a bodyweight comparable to that of C57-*C3H* mice.

The bodyweight of DBA mice raised by their biological mother was lower than that of DBA-*C3H*, but comparable to that of DBA-*AKR* (mother strain effect F(2,62) = 10.27 p<0.001 with DBA-*bio*≈DBA-*AKR* and DBA-*bio<*DBA-*C3H* p<0.001). This was seen both at weaning and in adulthood (age* mother strain F(2,62) = 0.16 p = ns).

### Cocaine intravenous self administration (figure 4)

**Figure 4:**
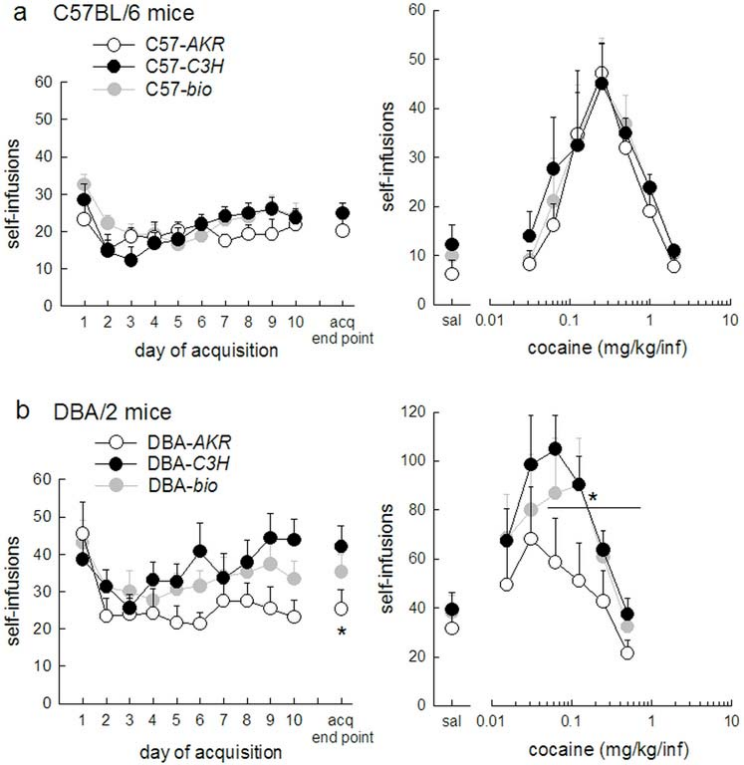
Impact of maternal environment on cocaine intravenous SA in C57 and DBA mice. Graphs show the number of self-infusions obtained in (a) C57 mice in acquisition at 1 mg/kg/infusion (left panel) and dose-response curve (right panel) (b) DBA mice in acquisition at 0.5 mg/kg/infusion (left panel) and dose-response curve (right panel). The symbols in the dose-response graph represent the last two days of stable intake on a dose. Behavior of the reference groups (-*bio*) is shown with grey lines and symbols. Horizontal lines show the data submitted to statistical analysis. Symbols represent mean±SEM. C57-*AKR* (white circles) *n* = 5, C57-*C3H* (black circles) *n* = 8, C57-*bio* (grey circles) *n* = 8, DBA-*AKR* (white circles) *n* = 8, DBA-*C3H* (black circles) *n* = 11, DBA-*bio* (grey circles) *n* = 10. * p<0.05 compared to DBA-*C3H.*

After ten days of training, both C57 (at 1 mg/kg/infusion) and DBA (at 0.5 mg/kg/infusion) acquired cocaine SA (figure 4 left panels). Animals from each experimental group showed a stable responding (±20%) over at least the last 3 days and reached the criteria of 75% responding for the active hole, indicating that they clearly discern the active from the inactive hole (hole effect C57-*AKR:* F(1,4) = 33.21 p<0.01, C57-*C3H:* F(1,7) = 9.27 p<0.05, C57-*bio*: F(1,7) = 22.4 p<0.01, DBA-*AKR:* F(1,7) = 12.37 p<0.01, DBA-*C3H:* F(1,10) = 63.16 p<0.001, DBA-*bio*: F(1,9) = 31.4 p<0.001, hole*day p = ns for all groups).

The two foster environments did not differentially affect acquisition of cocaine SA in C57 mice. Responding varied over the ten days of acquisition, but this variation was comparable for C57-*AKR* and C57-*C3H* animals (day effect F(9,99) = 3.87 p<0.001, day*mother F(9,99) = 1.61 p = ns). At the acquisition end point, C57-*AKR* and C57-*C3H* mice showed a similar number of self-infusions (t_1,11_ = −1.10 p = ns). On the contrary, in the DBA strain, acquisition was influenced by maternal environment. Responding varied over the ten days of acquisition, and this variation was dependent on mother strain (day effect F(9,153) = 2.82 p<0.01, day*mother F(9,153) = 2.12 p<0.05). At the acquisition endpoint, DBA-*AKR* mice showed a lower cocaine intake compared to DBA-*C3H* mice (t_1,17_ = −2.12 p<0.05).

Animals from each experimental group showed a significant dose-response to cocaine (figure 4 right panels), as they clearly distinguished the different doses (dose effect C57-*AKR* F(7,28) = 23.65; C57-*C3H* F(7,49) = 12.17; C57-*bio* F(7,49) =  16.07; DBA-*AKR* F(6,42) = 8.79; DBA-*C3H* F(6,60) = 14.79; DBA-*bio* F(6,54) = 15.79, all p<0.001). In this dose-response test however, DBA mice still showed important responses to the lower and less reinforcing doses of cocaine as well as to saline infusions. This ‘deficit’ of extinction resulted in a large variation for these doses. For this reason, only group differences in the descending limb of the dose-response curve (4 highest doses) were analyzed. Similarly to acquisition, maternal environment did not affect dose-response in C57 mice, but it did in DBA mice. C57 animals raised by an AKR or a C3H mother showed a similar dose-response curve for cocaine self-infusions (mother effect F(1,11) = 1.00 p = ns, dose*mother F(3,33) = 1.50 p = ns). On the contrary, DBA mice raised by an AKR mother showed a downward shift in the dose-response curve as compared to mice raised by a C3H mother (mother effect F(1,17) = 4.6 p<0.05, dose*mother F(3,51) = 0.45 p = ns).

SA behavior of C57-*bio* mice (represented by the grey lines in figure 4a) was similar to that of C57 mice raised by either foster mother. This was seen in the variation in intake during acquisition (day effect F(9,162) = 6.12 p<0.001, day*mother F(18,162) = 1.40 p = ns) as well as the intake at the acquisition endpoint (mother effect F(2,18) = 0.73 p = ns), and in the dose-response test (mother effect F(2,18) = 0.55 p = ns, dose*mother F(6,54) = 0.72 p = ns)

DBA-*bio* mice (represented by the grey lines in figure 4b) showed intermediate levels of responding in SA, more closely resembling the responding of DBA-*C3H* mice in the dose-response. Although visually apparent, these differences were not statistically significant (day effect in acquisition F(9,234) = 4.46 p<0.001, day*mother F(18,234) = 1.34 p = ns, mother effect at acquisition endpoint F(2,26) = 2.42 p = ns, mother effect in dose response F(2,26) = 2.45 p = 0.11 (ns), dose*mother F(6,78) = 0.57).

### Elevated plus-maze and forced swim test (figure 5)

**Figure 5:**
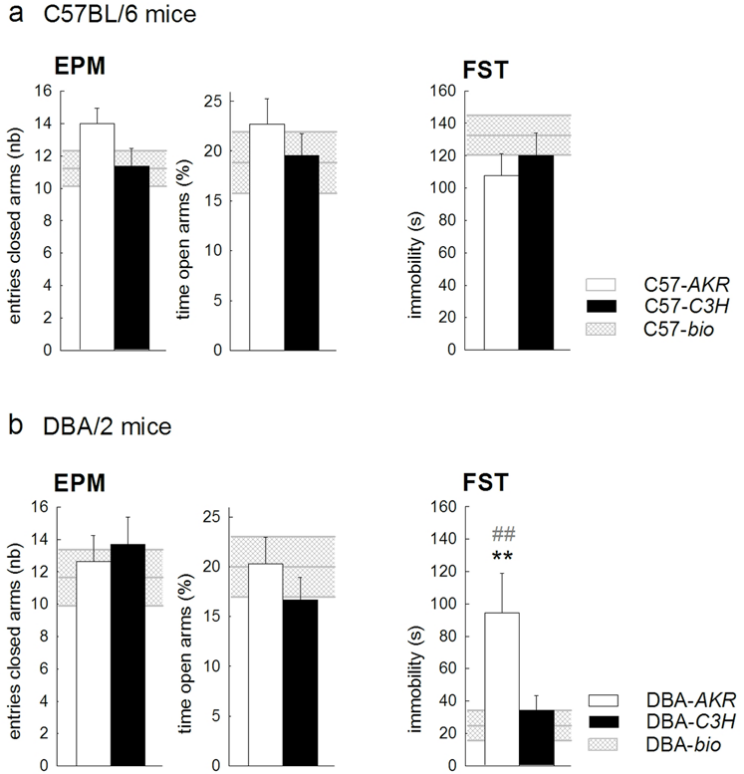
Impact of maternal environment on anxiety- and depression-related behaviors in (a) C57 and (b) DBA mice. *EPM:* Graphs show the total number of entries into the closed arms, an index of exploration (left panels) and the percentage of time spent on the open arms, a measure of anxiety (right panels). *FST:* Graphs show total immobility time in a 6 min test, a measure of depression-related behavior. Behavior of the reference groups (-*bio*) is shown as horizontal hatched bands. Bars represent mean±SEM. C57-*AKR* (white bars) *n* = 12, C57-*C3H* (black bars) *n* = 14, C57-*bio* (hatched bands) *n* = 14, DBA-AKR (white bars) *n* = 8, DBA-C3H (black bars) *n* = 10, DBA-*bio* (hatched bands) *n* = 11. ** p<0.01 compared to DBA-*C3H*. ## p<0.01 compared to DBA-*bio*.

We measured both locomotor activity and anxiety behavior on the EPM. We used the number of entries into the closed arms as a measure of locomotor activity [29] and the percentage of time spent on the open arms as a measure of anxiety [30]. Comparing behavior in the EPM, it appeared that neither locomotor activity (figure 5 left panels), nor anxiety behavior (figure 5 middle panels) was influenced by the foster environments (mother effect F(1,40) = 0.35 and 1.89, both p = ns; mother strain*pup strain F(1,40) = 1.96 and 0.01, both p = ns for respectively entries closed arms and time open arms).

In contrast, C57 and DBA mice were differentially influenced by the two foster environments in immobility behavior in the FST (figure 5 right panels; mother strain*pup strain F(1,40) = 5.56 p<0.05). Indeed, DBA-*AKR* mice showed an increased duration of immobility compared to DBA-*C3H* mice (p<0.01), while both C57 groups showed a comparable behavior in this test.

Behavior of C57-*bio* animals (hatched bands in figure 5a) in these two tests was comparable to that of animals raised by either foster mother (mother effect in EPM entries closed arms F(2,37) = 1.46, EPM time open arms F(2,37) = 0.40, FST immobility F(2,37) = 0.61, all p = ns).

Behavior of DBA-*bio* animals (hatched bands in figure 5b) in the EPM was also comparable to that of animals raised by either foster mother (mother effect in EPM entries closed arms F(2,26) = 0.40, EPM time open arms F(2,26) = 0.55, both p = ns). Behavior of DBA-*bio* animals in the FST was comparable to behavior of DBA-*C3H* mice and at a lower level than that of DBA-*AKR* mice (mother effect F(2,26) = 6.48 p<0.01, with DBA-*bio<*DBA-*AKR* p<0.01 and DBA-*bio≈*DBA-*C3H).*

## Discussion

Epidemiological and clinical studies point a role for negative experiences in the early life as risk factors for the development of psychopathologies in adulthood. However, individuals would be differentially susceptible to the same environmental challenges depending on their genetic background. Our results suggest that such gene-environment interactions can also be observed in rodents. Adult mice, exposed as pups to distinct maternal environments, were examined in cocaine SA and measures for depression- and anxiety- related behaviors, two behavioral dimensions that are frequently associated with human drug abuse [26]. We report that the outcome of maternal environment for cocaine SA and depression-related behavior in the offspring depends on their genotype. Indeed, C57 mice exhibited similar behaviors when raised in two distinct maternal environments, i.e. a C3H or an AKR mother, indicating a relative resistance to early environmental factors. In contrast, DBA mice raised by an AKR mother significantly differed from DBA mice raised by a C3H mother in cocaine intake and in immobility in the FST, indicating sensitivity to early environmental factors. Anxiety behavior as measured in the EPM was not affected by maternal environment in either strain.

When considering the reference groups (mice raised by their biological mother), it appears that a foster environment *per se*, created by either an AKR or a C3H mother, did not influence behavior in C57 mice, as C57-*AKR*, C57-*C3H*, and C57-*bio* mice were comparable for all behavior tested. Regarding DBA mice, the foster environment created by a C3H mother did not induce behavioral changes as DBA-*C3H* and DBA-*bio* mice are largely comparable, but the foster environment created by an AKR mother did. This latter environment appears to induce a decrease in cocaine intake and an increase in a depression-related behavior.

The design of our study was based on an alteration of the maternal environment of two inbred strains of mice, C57 and DBA, which were fostered with non-related mother strains. Compared to a classical cross-foster experiment, where C57 and DBA mice would have exchanged mothers, our approach reveals two interesting new aspects. Firstly, it allowed testing the outcome of two extremely different maternal environments and secondly, it permitted to minimize potential confounding factors generated by cross-fostering experiments. In classical cross-fostering experiments (exchanging pups between two strains), not only the quality of the maternal environment is manipulated, but also the genetic background of the mother. That is, while the control group is raised by an isogenic mother (e.g. DBA pup raised by a DBA mother), the experimental group is raised by a non-isogenic mother (e.g. DBA pup raised by a C57 mother). Using our model, we were able to demonstrate that the maternal environment, rather than the fostering to a non-isogenic mother is determinant in producing gene-dependent differences in adult behavior.

A factor that could be responsible for the influence of maternal environment on psychopathology-related behaviors is the maternal behavior [31]. The two foster mothers used in this study exhibit very distinct maternal behaviors. AKR mothers showed less pup-oriented behaviors (pup licking and nursing posture), and more nest disturbing behavior (nest reorganizing) and thus can be considered to provide an impoverished maternal environment compared to C3H mothers. Interestingly, the amount of pup licking, which is a very important component of maternal behavior [31], of both C57 and DBA biological mothers was situated in-between the two foster mothers. This underlines the clearly distinct maternal behavior of the AKR and C3H mothers. Our results suggest that this difference in maternal behavior could have played a role in the appearance of behavioral differences in adult DBA mice. However, it should be noted that the maternal environment is a complex ensemble including general behavioral patterns of the mother, but also factors like milk constitution and mother-pup communication through odor cues and vocalizations.

Probably central to gene-environment interactions is that a given environment is not perceived and handled with in the same way by different individuals. The context of maternal environment adds another level of complexity, i.e. the fact that the given environment is not inert but potentially responsive. Indeed, resembling the human situation, the environment furnished by the mother is influenced by the mother-pup dyad. Thus, two pups with different genetic background exposed to the same mother (genetically identical), might generate different behaviors or stimuli which in turn might influence the maternal environment in terms of maternal behavior, milk constitution or vocalizations. For example, the DBA-*AKR* and C57-*AKR* dyads generated the same level of pup licking and nest reorganizing by AKR dams, but not the same level of nursing posture. It remains questioned whether the gene-environment interaction observed in the present study resulted from DBA pups being more sensitive than C57 pups to similar aspects of the foster environment or from differences in the foster environments created by the mother-pup dyad.

Although foster mothers showed less nursing posture with DBA pups compared to C57 pups, this does not necessarily mean that DBA pups are less fed. Indeed, regarding bodyweight differences, undernourishment does not seem to be responsible for the observed gene-environment interaction. In the first place, bodyweight of pups raised by biological DBA mothers (that showed more nursing posture compared to both foster mothers), is similar to bodyweight of DBA-*AKR* pups. Comparing the biological litters of C57 and DBA mice, we can see that the overall lower bodyweight of DBA mice is due to a strain difference, rather than a difference in nursing posture. In the second place, for bodyweight at weaning, both pup strains are affected in the same way by the foster environments. That is, pups raised by an AKR mother showed a lower bodyweight compared to pups raised by a C3H mother. At adult age, these bodyweight differences were still visible, although less pronounced. Interestingly, the influence of maternal environment on bodyweight was even stronger in C57 than in DBA mice.

Only a few studies investigated the genotype-dependent impact of the early life period on psychopathology-related behaviors in adulthood. To our knowledge, studies regarding drug intake only concern alcohol and support our findings. That is, when tested at the age of weaning, cross-fostering influenced alcohol intake in DBA, but not in C57 mice [32]. A recent study using selected rat lines showed a genotype-dependent impact of maternal separation on alcohol intake in rats [33]. Also anxiety- [34] and depression-related behaviors [35] were shown to be affected by cross-fostering in a genotype-dependent manner.

Interestingly, rodent models in this field resemble the human condition in two aspects. Firstly, a given genotype is not vulnerable or resistant to a given environment in all aspects of its behavior. We for example, showed that DBA are sensitive to the effect of cross-fostering on cocaine use and a depression-related behavior, but not on an anxiety-related behavior. Similarly to our observations, C57 mice are often reported to be resistant to the influence of early life manipulations on several aspects of adult behavior, including cognitive abilities, depression-related and schizophrenia-related behaviors [34], [36]–[38]. However, early life manipulations do influence aggressive behavior in this strain [39]. Secondly, a given genotype would not be vulnerable or resistant to all types of early life manipulations. In our study, DBA mice were susceptible to the effect of cross-fostering on a depression-related behavior, while others have shown that maternal separation or handling were ineffective in this strain [40]. In the same way, cross-fostering of the C57 to a BALB/c mother did not change anxiety-related behavior in the C57 mice [34], [41], while maternal separation did [39], [42], although the effects of maternal separation on anxiety might depend on the protocol used [43].

Our data suggest an opposite relation between a depression-related behavior and cocaine intake. The comorbidity between depression and cocaine abuse that is frequently described, appears contradictory with this finding. To explain comorbidity, the self-medication theory proposes that depressed patients seek for the specific effects of cocaine to relieve distress associated with depression [25]. However, the causal links between depression and cocaine use are controversial. It needs to be mentioned that cocaine abuse would rather be associated with bipolar depression rather than with unipolar depression [44]. Moreover, in comorbid patients, depression could rather be a consequence than a cause of cocaine abuse [45]. Indeed, depressive symptoms are frequently mentioned as a consequence of drug use or withdrawal [46], [47]. Furthermore, our observation of an opposite relation between a depression-related behavior and cocaine intake is in accordance with other rodent studies. A decreased sensitivity to reward was found in animals in which a depressive-like behavior was induced either by exposure to chronic mild stress (CMS) [48] or olfactory-bulbectomy (OBX) [49]. Thus, in rats, CMS induces an increased immobility in the FST associated with a decrease in reward sensitivity as measured by increased intracranial self-stimulation threshold, decreased sucrose consumption, decreased preference for alcohol, decreased sexual behavior and decreased amphetamine and morphine rewarding effects [48]. Similarly, OBX, that exhibits a high degree of neurochemical similarity to depression, induces a decreased sensitivity to reward as shown by a decreased sexual behavior [50], an increased intracranial self-stimulation threshold [51] and a reduced cocaine place preference [52].

In conclusion, we showed for the first time that gene-environment interactions during the early life period can affect cocaine use in adulthood. We further demonstrated an association with a depression-related behavior. Our experimental approach could contribute to the identification of the psychobiological factors determining the susceptibility or the resilience of certain individuals to develop psychopathologies.
